# Primary care physicians' use of family history for cancer risk assessment

**DOI:** 10.1186/1471-2296-11-45

**Published:** 2010-06-03

**Authors:** Brian S Flynn, Marie E Wood, Takamaru Ashikaga, Alan Stockdale, Greg S Dana, Shelly Naud

**Affiliations:** 1 Department of Family Medicine, College of Medicine, University of Vermont, Burlington Vermont, 05405, USA; 2 Office of Health Promotion Research, College of Medicine, University of Vermont, Burlington Vermont, 05405, USA; 3 Department of Medicine, College of Medicine, University of Vermont, Burlington Vermont, 05405, USA; 4 Department of Medical Biostatistics, College of Medicine, University of Vermont, Burlington Vermont, 05405, USA; 5 Education Development Center, Inc., Newton Massachusetts, 02458, USA

## Abstract

**Background:**

Family history (FH) assessment is useful in identifying and managing patients at increased risk for cancer. This study assessed reported FH quality and associations with physician perceptions.

**Methods:**

Primary care physicians practicing in two northeastern U.S. states were surveyed (n = 880; 70% response rate). Outcome measures of FH quality were extent of FH taken and ascertaining age at cancer diagnosis for affected family members. Predictors of quality measured in this survey included: perceived advantages and disadvantages of collecting FH information, knowledge of management options, access to supportive resources, and confidence in ability to interpret FH.

**Results:**

Reported collection of information regarding second degree blood relatives and age of diagnosis among affected relatives was low. All hypothesized predictors were associated with measures of FH quality, but not all were consistent independent predictors. Perceived advantages of taking a family history, access to supportive resources, and confidence in ability to identify and manage higher risk patients were independent predictors of both FH quality measures. Perceived disadvantages of taking a family history was independently associated one measure of FH quality. Knowledge of management options was not independently associated with either quality measure.

**Conclusions:**

Modifiable perception and resource factors were independently associated with quality of FH taking in a large and diverse sample of primary care physicians. Improving FH quality for identification of high risk individuals will require multi-faceted interventions.

## Background

Cancer risk assessment can be used in primary care to identify patients who may benefit from risk management strategies. A family history is a relatively simple and accurate method of stratifying risk for several major cancers [1-6].

Recommendations from national consensus groups support the case for systematic use of family history to assess cancer risk. The U.S. Preventive Services Task Force (USPSTF) statement on colorectal cancer screening considers patients with a first degree relative diagnosed before age 60 to be at increased risk [7]. Characterization of increased risk as moderate or high requires information on second degree relatives. The moderate risk group is recommended for accelerated screening and the higher risk group may be candidates for assessment of hereditary disease [8]. Breast cancer risk stratification recommendations follow a similar pattern [9]. Use of family history information for cancer risk assessment is recommended by other professional organizations [10-12].

Although family histories are collected routinely, gaps that reduce their value for risk assessment have been reported. Data on second degree relatives and age of diagnosis are often missing and data are updated infrequently [2,13-19]. Barriers to more effective use of family histories have included time costs, low confidence in ability to effectively collect and utilize data, and lack of clear guidelines [4,20-25].

This study was designed to identify predictors of family history quality among primary care physicians as a basis for developing strategies to improve cancer risk assessment and management. Social Cognitive Theory (SCT) was used as a conceptual framework for developing hypotheses and measures of key variables [26,27]. This model describes three major types of influence on behavior: intrapersonal factors, such as beliefs about the results of performing a behavior (outcome expectations), and beliefs about ability to perform the behavior (confidence or self-efficacy); perceived environment factors, such as peer norms or access to supportive resources related to the behavior; and behavioral factors such as past behavior and skills needed to perform the behavior.

Hypotheses derived from this conceptual model stated that physicians would be more likely to take higher quality family histories if reporting: higher positive and lower negative beliefs about the value of identifying familial risks; greater knowledge of management options for higher risk patients; higher levels of resources to assist in using family history information; and higher levels of confidence in ability to identify and manage familial cancer risk.

## Methods

The study was based on data collected through surveys of primary care physicians in the northeastern United States during 2005. Three health care organizations provided lists of physicians practicing general internal medicine, family medicine, and gynecology in urban, suburban, and rural communities. The survey was conducted with up to four mailings; physicians responding or opting-out were not sent additional mailings. Procedures were approved by institutional review boards at the University of Vermont and collaborating organizations.

Predictor and outcome measures were developed using qualitative interviews with 20 physicians [28] and consultation with an expert panel and other primary care physicians. Measures were refined using principal components factor analyses to confirm that intercorrelated groups of items comprised valid measures [29]; internal consistency reliability was estimated using Cronbach's alpha. Five theory-based predictors and two outcome variables were assessed. The survey instrument is shown in Additional file 1.

### Predictor measures

Advantages of Taking Family History. A seven item measure assessed expectations that taking a family history would be advantageous (e.g., "basis for providing preventive guidance to patients"). Four response options for each item ranged from "disagree" (1) to "agree" (4). The internal consistency reliability coefficient was alpha = .72.

Disadvantages of Taking Family History. A seven item measure assessed expectations that taking a family history would be disadvantageous (e.g., "difficult to interpret risk based on family history"). Four response options ranged from "disagree" (1) to "agree" (4). Alpha = .83.

Risk Management Knowledge. A thirteen item measure assessed knowledge of options for managing a patient identified as at risk for cancer because of family history. Seven items described possible management strategies for breast cancer risk based on family history (e.g., "increased frequency of screening"); six similar items focused on familial risk of colon cancer (e.g. "earlier initiation of screening"). Five response options ranged from "unlikely" (1) to "likely" (5). Alpha = .74.

Supportive Resources. A seven item measure assessed perceived levels of support for using family history information to assess risk. Respondents were asked to rate their use of possible resources (e.g., "professional organization guidelines"). Five response options ranged from "never" (1) to "always" (5). Alpha = .74.

Confidence. A four item measure assessed confidence that respondents could identify and manage patients at risk of cancer due to family history. For both breast and colon cancer they were asked to rate confidence that "you can identify a patient who may be at increased risk" and that "you can effectively manage a patient who is at increased risk" based on family history. Eleven response options ranged from "not at all confident" (0) to "completely confident" (10). Alpha = .82.

These predictor variables were moderately correlated with one another in the expected directions, indicating both content validity and the independence of the constructs measured. Advantages of Taking Family History, for example, was negatively correlated with Disadvantages of Taking Family History (Pearson correlation r = -.40) and positively correlated with Risk Management Knowledge (r = .27), Supportive Resources (r = .25), and Confidence (r = .26).

### Outcome measures

Family History Taking Extent. A five item measure assessed how often respondents included specific first and second degree family members in a family history. Listed relatives included parents, children, siblings, grandparents, and aunts and uncles. Five response options for each item ranged from "never" (1) to "always" (5). Alpha = 0.68.

Age of Diagnosis Determination. A single item assessed the frequency with which a physician collected the age of diagnosis for a family member's disease. The question asked "when you collect family history information, how often do you include age of diagnosis?" Five response options for this item ranged from "never" (1) to "always" (5).

The two outcome variables were moderately and positively correlated (r = .22).

### Statistical analyses

Preliminary analyses examined distributions and assessed impact of respondent characteristics on outcome variables to determine the need to include these in the main analyses [30]. For the hypothesis tests, each potential predictor was entered into a multiple regression analysis for each of the two quality measures, controlling for variables representing geographic location of the physician's practice and specialty. Relative strength of relationships between predictor and outcome variables was assessed by analyses that included all five predictor measures and the two control variables with each of the two quality measures as outcomes.

## Results

A total of 880 surveys were returned by 1265 physicians to whom forms were mailed and not returned because of non-delivery (70% response rate overall; response rates varied somewhat by specialty: family medicine 75%, general internal medicine 65%, and gynecology 84%). Median year of graduation from medical school was 1984. Sixteen were African American, 62 were Asian or Pacific Islanders, 787 were Caucasian, and 12 reported another racial category; 18 reported Hispanic ethnicity; and 314 were female. Participants included 479 whose practice was general internal medicine, 298 in family medicine, and 90 in gynecology. Representation of specialties varied by area with relatively more family medicine physicians in rural areas and more internists in suburban and urban areas (Table 1). The median number of physicians in practice groups was 5. Practice groups were not affiliated with an academic medical center for 473, were affiliated with a center for 272, and were located within a center for 128.

**Table 1:** Physician practice specialties by geographic location.

	Urban	Suburban	Rural	Total
Family Medicine	24 19%	77 24%	197 47%	298 34%

Internal Medicine	86 68%	239 74%	154 37%	479 55%

Gynecology	16 13%	6 2%	68 16%	90 11%

Total	126 100%	322 100%	419 100%	867 100%

Chi-Square (4 d.f.) = 126.1; p < .001

Participants reported highest frequency of collecting family history data at first or second visit of a new patient (mean frequency score = 4.7 on the 1-5 scale). Lower frequencies of family history data collection were reported for periodic examinations (mean score = 4.0), chronic disease visits (3.1), and acute care visits (2.1). Substantial differences were reported for collection of information about first, second, and third degree relatives of patients (Table 2). At one extreme, nearly all reported that they "always" collected history information about parents. Smaller proportions often collected information about second and third degree relatives. Frequency of collecting details about an affected relative also varied. Most physicians (84%) "always" collected cancer diagnosis information. Age of diagnosis information was collected less frequently: responses ranged from "never" (1) for 1.1%, to (2) for 6.5%, (3) for 24.1%, (4) for 36.9%, and "always" (5) for 31.3%. Details of treatment for the affected family member(s) were collected rarely.

**Table 2:** Physician reported frequency of family history taking by types of family relationships (n = 867)

	% **never** 1	% 2	% 3	% 4	% **always** 5
First Degree					

Parents	0.3	0.5	0.8	2.4	96.0

Siblings	0.5	0.9	2.7	8.5	87.4

Children	2.1	5.8	9.1	12.0	71.0

Second Degree					

Grandparents	2.3	5.6	17.0	26.7	48.5

Aunts/Uncles	4.5	15.0	35.5	25.3	19.6

Third Degree					

Nieces/Nephews	21.7	37.9	25.1	9.0	6.4

Cousins	25.7	37.3	22.7	8.0	6.3

These outcomes differed slightly but not significantly by practice location. Differences in FH Taking Extent by practice specialty were significant (p = .05) but small with the mean scores for family medicine practitioners at 22.0, gynecology at 21.6, and internal medicine at 21.4. No significant differences by specialty were found for the Age of Diagnosis outcome.

### Predictors of family history-taking quality

Results for the Advantages measure showed a mean score of 3.5 (on the four point scale from 1 = disagree to 4 = agree) indicating a high level of primary care physician agreement with the stated advantages of taking a family history; the mean score for the Disadvantages measure was 2.0, indicating a moderate level of physician disagreement with the stated disadvantages of taking a family history. The Management Knowledge measure had a mean score of 3.3 on a 1-5 scale indicating a moderate level of likely use of these options. The Supportive Resources mean score was 2.5 on a 1-5 scale, indicating relatively infrequent use of these resources. The mean Confidence score was 7.1 on a 0-10 scale indicating moderate confidence.

A small difference was found by practice location for Advantages (p = .01) with mean scores for suburban physicians at 3.3, and for rural and suburban physicians at 3.2. More notable differences in predictor scores were found by practice specialty. Knowledge of Treatment Options was higher (p < .01) among gynecology physicians (mean = 3.4) than among internal medicine or family medicine practitioners (3.2). Confidence in identifying and managing patients at higher risk of cancer because of family history, on the other hand, was lower (p < .01) among gynecology physicians (6.7) than among family medicine (7.3) or internal medicine (7.1) practitioners.

### Associations between predictors and outcomes

Analyses testing the study hypotheses yielded a consistent pattern of significant associations between predictors and outcomes (Table 3). The correlation between Advantages and Family History-Taking Extent was r = 0.25 (P < .001), indicating that physicians who agreed with statements describing advantages of family history-taking were more likely to take an extensive history. Conversely, the negative correlation between Disadvantages and Family History-Taking Extent (r = -.20; P < .001) showed that physicians who agreed with disadvantage statements were less likely to take an extensive history. Positive relationships also were found between Family History-Taking Extent and other hypothesized predictors. These relationships were tested with the second outcome, Age of Diagnosis Determination, with similar results (Table 3).

**Table 3:** Association of each hypothesized predictor with family history-taking quality indicators (n = 867).

	**Outcomes**			
	
**Predictors**	FH Taking Extent		Age of Diagnosis Determination	
	
	Slope (S.E.)	Partial Correlation	Slope (S.E.)	Partial Correlation
Advantages	1.81 (.25)	.25***	0.72 (.08)	.30***

Disadvantages	-1.02 (.17)	-.20***	-0.27 (.05)	-.17***

Management Knowledge	0.96 (.19)	.18***	0.31 (.06)	.17***

Supportive Resources	1.02 (.18)	.20***	0.44 (.06)	.26***

Confidence	0.35 (.07)	.17***	0.16 (.02)	.24***

*** p < .001

### Relative strength of predictors

All five predictors and the two control variables were entered simultaneously into a multiple regression analysis with Family History-Taking Extent as the outcome variable. Four of the five predictors had significant independent effects (Table 4). The strongest predictor was Disadvantages (β = -0.13), followed by Supportive Resources (β = 0.109), Advantages (β = 0.097), and Confidence (β = 0.085). The Management Knowledge measure did not have a significant independent relationship with Extent. A similar analysis using Age of Diagnosis Determination as the outcome yielded similar results with Advantages, Supportive Resources, and Confidence as significant independent predictors; Disadvantages and Management Knowledge were not significant predictors for this outcome. The Advantages, Supportive Resources, and Confidence measures were independent predictors for both of the quality indicators. Management Knowledge was not independently associated with either outcome.

**Table 4:** Simultaneous tests of association of hypothesized predictors with family history-taking quality indicators (n = 867).

	**Outcomes**			
	
**Predictors**	FH Taking Extent		Age of Diagnosis Determination	
	
	parameter estimate (s.e.)	standardized coefficient	parameter estimate (s.e.)	standardized coefficient
Advantages	0.72 (.30)	0.097*	0.50 (.10)	0.202***

Disadvantages	-0.63 (.19)	-0.130***	-0.01 (.06)	-0.009

Management Knowledge	0.35 (.22)	0.064	0.04 (.07)	0.020

Supportive Resources	0.56 (.20)	0.109**	0.26 (.06)	0.152***

Confidence	0.17 (.07)	0.085*	0.096 (.02)	0.147***

*** p < 0.001, **p < 0.01, *p < 0.05

### Salient barriers and facilitators

Mean scores for individual items in selected predictor measures were ranked to identify more specific barriers and facilitators. The most strongly endorsed Advantage items were: "better understand risk of future disease" (mean score of 3.9 on the 1-4 scale); "better rationale for screening modalities" (3.8); "basis to provide preventive guidance to patients" (3.7); and "better address patient's concerns" (3.5). Disadvantage items most likely to elicit agreement were: "patients often have limited knowledge of family history" (mean score of 2.5 on the 1-4 scale); "less important than other office visit tasks" (mean = 2.2); "difficult to interpret risk based on family history" (2.0); and "difficult to communicate risk based on family history" (1.9). The supportive resources utilized most often included: "professional organization guidelines" (mean score of 3.4 on the 1-5 scale); "refer to a specialist" (3.2); "discuss with a specialist" (3.0); "other published guidelines" (3.0); and "on-line resources" (2.9).

## Discussion

This survey of primary care physicians provides information about self-reported family history-taking behaviors, views on using family history for cancer risk assessment and management, and associations between these perceptions and behaviors from a large sample of primary care physicians. The sample was based on comprehensive rosters of physicians in selected locations. The range of community settings and specialties and the high response rates provided an unusually complete and representative portrait of primary care physicians in these areas. Results from this diverse sample of physicians are generally consistent with findings from more focused studies, providing additional confidence in the generalizability of the findings.

Reported family history taking among these physicians was concentrated on first degree relatives and specific diagnoses, but not second degree relatives or age of diagnosis for those affected. Family history data were often collected at new patient visits for periodic health exams, but were not often updated. These results were consistent with interviews of primary care physicians suggesting that family histories often were collected as part of the development of a general understanding of the patient's situation [28]; systematic use of family history to identify and manage cancer risk was rarely reported [4,13,25,31].

All of the hypothesized physician perceptions were significantly associated with reported quality of family history-taking when entered separately into analyses (Table 3). When all five potential predictors were entered into analyses simultaneously, however, several of these demonstrated independent associations with both outcomes (Table 4), indicating that these variables better explained variation in the outcomes than perception measures which demonstrated associations only when entered separately. Perceived Advantages of taking a family history, perceptions of Supportive Resources to facilitate use of family history information, and Confidence in interpreting and using family history results for management of cancer risk independently predicted both measures of family history taking quality. The consistency and statistical significance of these associations suggested that these results provide useful guidance for design of interventions to improve utilization of this cancer risk assessment strategy. Perceived Disadvantages was a strong independent predictor of FH Taking Extent, but was not independently associated with Age of Diagnosis Determination. Management Knowledge was not independently associated with either outcome.

The results provided an encouraging picture of high receptivity to use of family history for cancer risk assessment. In general, participants tended to agree with statements describing Advantages and disagree with statements describing Disadvantages of taking a family history, consistent with other reports of value placed on the strategy of using family history to identify cancer risk [20-22]. Both of these measures were relatively strong independent predictors of one or another of the two outcome measures. The strength of these associations with the family history quality suggested that small differences in these perceptions could have a large impact on clinical behaviors.

The importance of availability and usability of resources focused on collection and interpretation of family history information is highlighted by the independent association of the Supportive Resources measure with both quality outcome measures. The most frequently utilized among the seven resources listed was professional organization guidelines followed by referral or consultation, other published guidelines, and on-line resources. These results reflected a relatively high regard for guidelines endorsed by respected sources relative to other types of resources. This finding is consistent with needs for more specific guidelines and other readily accessible supportive resources as suggested by other investigators [19-21,25].

Key issues affecting increased levels of guideline use may include usability of guideline statements and related resources within the context of an office visit [3,6,19,32,33]. Current guidelines for use of family history in cancer risk assessment are not organized in a consolidated statement that can be easily implemented in primary care practice. Some attempts have been made to integrate the major consensus statements into more usable summaries that directly link family history data to risk assessment and management [5,34].

Confidence was independently associated with both measures of family history quality as hypothesized. Overall scores indicated moderate levels of confidence, similar to relatively low or moderate levels of confidence identified previously [4,20-22]. More focused family history resources and systems built on succinct consolidated guideline statements concerning cancer risk assessment and management may increase confidence levels.

The hypothesized Cancer Risk Management Knowledge predictor was not significantly associated with either quality indicator. It is possible that the links between family history information and use of management options for patients at higher risk were not clear for some respondents, despite strong endorsement of the overall rationale. This would be an important gap since expanded risk management opportunity is a primary motivation for the investment of effort required for more systematic family history data collection.

Several important barriers to collection and utilization of family history information emerged. These included patient knowledge of their family history, relative value of family history collection compared to other office visit tasks (possibly an indication of time efficiency concerns), and difficulties of interpreting and communication risk. These are substantial barriers but they can be addressed by new strategies. The rapid adoption of electronic health records in some countries presents new opportunities for development of systems to facilitate family history data collection, risk assessment, and consideration of management options. These new resources may provide for more time-efficient processes and a greater role for patients in collecting and recording family history information.

This study has several limitations. Although results were consistent with causal connections between self-reported perceptions and indicators of family history quality, this was a cross-sectional study testing correlational hypotheses. The outcome measures may not accurately reflect family history-taking behavior, with the most likely bias being reporting of more positive behavior. The modest amount of variance explained indicated the potential importance of variables not measured. Lastly, the conclusions may have limited generalizability as the study surveyed physician practicing in targeted communities of one region in the United States.

## Conclusions

These results suggest that family history information could be used more systematically to identify patients who may benefit from cancer risk assessment. Clinical practice of cancer risk assessment could be improved by changes in the quality of routinely collected family history data, focusing on inclusion of first and second degree relatives, age at which a cancer is diagnosed and periodic updating.

Although national organizations endorse use of family history data for this purpose and physicians supported the approach, several issues appeared to impede more complete implementation of this cancer risk management strategy. Efficient collection of family history information may require a better-defined role for patients in providing baseline and updated information as envisioned in the U.S. Surgeon General's initiative [35]. Patient efforts may need to be focused and complemented by more systematic health maintenance protocols for cancer risk assessment. Low levels of use of current resources for interpreting and utilizing family history data suggest a need for development of more efficient and accessible methods designed to integrate with the flow of work in typical office visits [4,23,32,36].

## Competing interests

The authors declare that they have no completing interests.

## Authors' contributions

BF participated in the design, survey development and implementation, and data analysis, and drafted the manuscript. MW participated in the design, survey development, sample recruitment, data analysis, and manuscript writing, and provided overall leadership. AS contributed to the survey development. GD participated in survey development and implemented the survey. TA contributed to the design and led the data analysis. SN contributed to the data analysis. All authors read and approved the final manuscript.

## Pre-publication history

The pre-publication history for this paper can be accessed here:

http://www.biomedcentral.com/1471-2296/11/45/prepub

## Supplementary Material

Additional file 1 **Family History and Cancer Risk Assessment - Physician Survey**. Six page self-completion mail survey Click here for file
