# Supplementary material for: Primary care physicians' use of family history for cancer risk assessment
Source: BMC Fam Pract. 2010 Jun 3;11:45. doi: 10.1186/1471-2296-11-45 (PMC2898682; doi:10.1186/1471-2296-11-45)
Supplement: Additional file 1 — Family History and Cancer Risk Assessment - Physician Survey. Six page self-completion mail survey [file 1471-2296-11-45-S1.PDF]

Please choose from the responses offered. Comments can be written anywhere on the form.

1. The main focus of your practice could best be described as:

    1. Family Practice   2. Internal Medicine
    3. OB/GYN   4. Other (please specify): ________________________

---

2. The approximate number of physicians in your practice group is: ____________________

---

3. The Zip Code for your usual practice location is: ____________________

---

4. Your practice group is:

    1. within an academic medical center
    2. affiliated with an academic medical center
    3. not affiliated with an academic medical center

---

5. Your year of graduation from medical school was: __ __ __ __

---

6. Your sex is: 1. female   2. male

---

7. Your racial background is… (choose any applicable categories):

    1. African American/Black   2. Asian/Pacific Islander   3. Caucasian
    4. Native American   5. Other ______________________

---

8. Your ethnic background is: 1. Hispanic   2. not Hispanic

Questions 9-14 are about family history-taking in general.

9. What method(s) do you use to obtain family history information from your patients?

a. patient-completed form	**NO**	**YES**
b. physician interview	**NO**	**YES**
c. nurse interview	**NO**	**YES**

d. other method:___________________________________

---

10. How often do you collect and record family history information from your patients in the following situations?

	never				always
a. new patient - first or second visit	1	2	3	4	5
b. urgent visit (e.g. URI)	1	2	3	4	5
c. chronic disease management visit	1	2	3	4	5
d. periodic exam visit	1	2	3	4	5

---

11. How often do you include these types of relatives when you collect family history information from your patients?

	never				always
a. parents	1	2	3	4	5
b. children	1	2	3	4	5
c. siblings	1	2	3	4	5
d. grandparents	1	2	3	4	5
e. aunts and uncles	1	2	3	4	5
f. nieces and nephews	1	2	3	4	5
g. cousins	1	2	3	4	5

---

12. When you collect family history information, how often do you include the following details?

	never				always
a. diagnosis	1	2	3	4	5
b. age of diagnosis	1	2	3	4	5
c. treatment	1	2	3	4	5

---

13. The following statements summarize possible <u>reasons to collect detailed family history information</u>. Choose the response closest to your opinion.

	disagree			agree
a. assist in interpreting current symptoms	1	2	3	4
b. better address patient's concerns	1	2	3	4
c. better understand risk of future disease	1	2	3	4
d. better rationale for screening schedules or modalities	1	2	3	4
e. basis for providing preventive guidance to patients	1	2	3	4
f. provides guidance for considering chemoprevention	1	2	3	4
g. this is a standard of care	1	2	3	4

14. The following statements summarize possible <u>reasons to NOT collect detailed family history information</u>. Choose the response closest to your opinion.

	disagree			agree
a. less important than other office visit tasks	1	2	3	4
b. difficult to interpret risk based on family history	1	2	3	4
c. difficult to communicate risk based on family history	1	2	3	4
d. scientific basis for using this information not well-established	1	2	3	4
e. limited practical applications of family history information	1	2	3	4
f. patients often have limited knowledge of family history	1	2	3	4
g. identifying elevated risk may cause unproductive anxiety	1	2	3	4

The remaining questions are about family history and cancer.

15. How often do you use any of these resources to <u>assess a patient's cancer risk based on family history</u>?

	never				always
a. discuss with a colleague	1	2	3	4	5
b. discuss with a specialist	1	2	3	4	5
c. refer to a specialist	1	2	3	4	5
d. professional organization guidelines	1	2	3	4	5
e. insurance company guidelines	1	2	3	4	5
f. other published guidelines	1	2	3	4	5
g. on-line resources (e.g. UpToDate)	1	2	3	4	5

h. other resources used:____

16. Rate your level of confidence that you can identify a patient who may be at increased risk of breast cancer based on her family history. (Mark anywhere on the 0-10 scale.)

0	1	2	3	4	5	6	7	8	9	10
**not at all confident**										**completely confident**

---

17. Rate your level of confidence that you can identify a patient who may be at increased risk of colon cancer based on his or her family history.

0	1	2	3	4	5	6	7	8	9	10
**not at all confident**										**completely confident**

---

18. Which of the following recommendations would you be likely to make for a patient who appeared to be at high risk for breast cancer based on your assessment of her family history?

	**unlikely**				**likely**
a. earlier initiation of screening	1	2	3	4	5
b. increased frequency of screening	1	2	3	4	5
c. immediate referral to specialist	1	2	3	4	5
d. lifestyle modification	1	2	3	4	5
e. chemoprevention (e.g. tamoxifen)	1	2	3	4	5
f. prophylactic surgery	1	2	3	4	5
g. referral for genetic counseling	1	2	3	4	5

h. other likely recommendation:________________________________

---

19. Which of the following recommendations would you be likely to make for a patient who appeared to be at high risk for colon cancer based on your assessment of his or her family history?

	**unlikely**				**likely**
a. earlier initiation of screening	1	2	3	4	5
b. increased frequency of screening	1	2	3	4	5
c. immediate referral to specialist	1	2	3	4	5
d. lifestyle modification	1	2	3	4	5
e. chemoprevention (e.g. NSAID)	1	2	3	4	5
f. referral for genetic counseling	1	2	3	4	5

g. other likely recommendation:_________________________________

20. Rate your level of <u>confidence that you can effectively manage a patient who is at increased risk of breast cancer</u> based on your assessment of her family history.

0	1	2	3	4	5	6	7	8	9	10
**not at all confident**										**completely confident**

---

21. Rate your level of <u>confidence that you can effectively manage a patient who is at increased risk of colon cancer</u> based on your assessment of his or her family history.

0	1	2	3	4	5	6	7	8	9	10
**not at all confident**										**completely confident**

---

22. Have you ever referred a patient for genetic counseling or testing because of questions raised by a <u>family history of cancer</u>? **NO** **YES**

---

23. Have you ever referred a patient for genetic counseling or testing because of questions raised by <u>any other family history issue</u>? **NO** **YES**

---

24. Have you ever referred a patient for genetic counseling or testing primarily because of that <u>patient's request</u>? **NO** **YES**

---

25. The following statements summarize possible <u>reasons to refer patients for genetic counseling or testing</u> because of a suggestive family history of cancer. Choose the response closest to your opinion.

	**disagree**			**agree**
a. potentially beneficial to patients	**1**	**2**	**3**	**4**
b. potentially relevant to clinical decisions	**1**	**2**	**3**	**4**
c. counseling and testing services are readily accessible	**1**	**2**	**3**	**4**
d. potentially productive cancer control strategy	**1**	**2**	**3**	**4**
e. possible liability if do not fully evaluate for hereditary cancer syndrome	**1**	**2**	**3**	**4**
f. some patients strongly express a need for genetic testing	**1**	**2**	**3**	**4**
g. referral is the standard of care when history is suggestive	**1**	**2**	**3**	**4**
h. a useful genetic test is available for the disease of interest	**1**	**2**	**3**	**4**

26. The following statements summarize possible reasons to NOT refer patients for genetic counseling or testing because of a suggestive family history of cancer. Choose the response closest to your opinion.

	**disagree**			**agree**
a. uncertain value of results	1	2	3	4
b. poor quality of feedback to referring physician	1	2	3	4
c. counseling and testing services are not readily accessible	1	2	3	4
d. informative tests are available for very few diseases	1	2	3	4
e. patient reluctance	1	2	3	4
f. high financial cost to patient	1	2	3	4
g. possibility of insurance discrimination	1	2	3	4
h. genetic counseling and testing are not now standard care	1	2	3	4

---

27. How useful might each of the following be in augmenting your ability to assess cancer risk associated with family histories?

	**unlikely to be useful**				**likely to be useful**
a. education programs at national meetings	1	2	3	4	5
b. education programs at local meetings	1	2	3	4	5
c. office-based educational program	1	2	3	4	5
d. education programs on CD/DVD	1	2	3	4	5
e. published guidelines	1	2	3	4	5
f. peer-reviewed journal articles	1	2	3	4	5
g. other published reference materials	1	2	3	4	5
h. succinct protocols	1	2	3	4	5
i. on-line guidelines	1	2	3	4	5
j. on-line reference sources (e.g. UpToDate)	1	2	3	4	5
k. local computer software with decision support	1	2	3	4	5

l. other useful educational or reference resources: ____________________________

Thank you for completing this survey. Please return it in the addressed stamped envelope to

[investigator address]

Send the addressed postcard separately when your questionnaire is mailed.
